# Supplementary figures and images for: Cross-reactive antibodies after SARS-CoV-2 infection and vaccination
Source: eLife. 2021 Nov 23;10:e70330. doi: 10.7554/eLife.70330 (PMC8610423; doi:10.7554/eLife.70330)

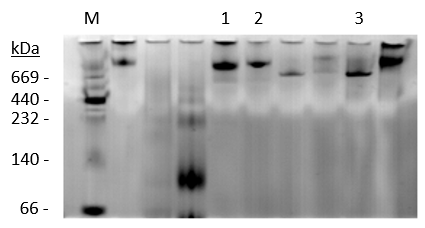

Supplement: Figure 2—figure supplement 5—source data 1. — The numbers match with the numbers in Figure 2—figure supplement 5: (M) Marker HMW-Native Protein Mixture; (1) SARS-CoV spike; (2) MERS-CoV spike; (3) hCoV-229E spike; (4) hCoV-OC43 spike; (5) SARS-CoV-2 spike; (6) hCoV-HKU1 spike; and (7) hCoV-NL63 spike. [file elife-70330-fig2-figsupp5-data1.zip › Gel 1 labeled.png]

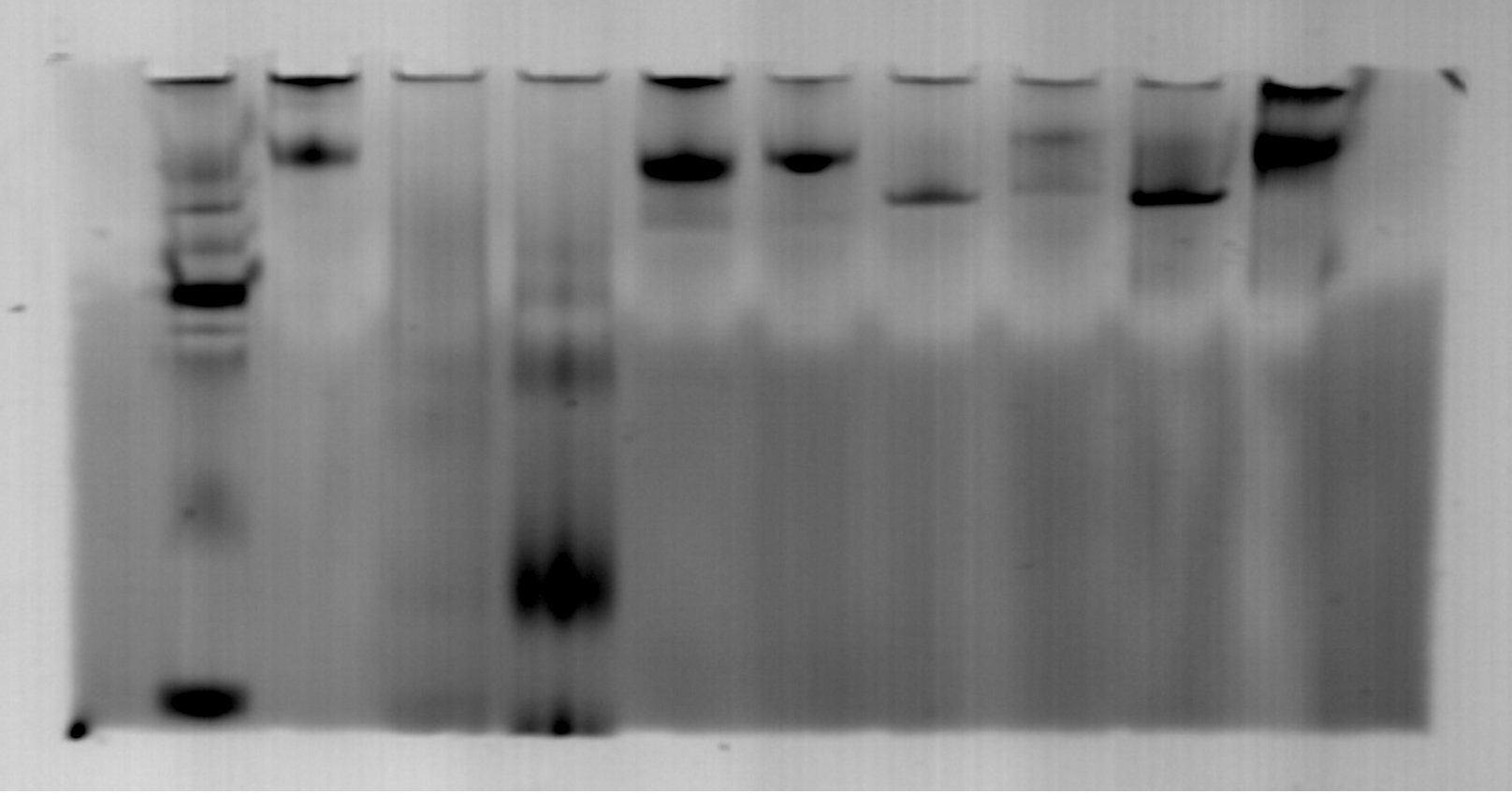

Supplement: Figure 2—figure supplement 5—source data 1. — The numbers match with the numbers in Figure 2—figure supplement 5: (M) Marker HMW-Native Protein Mixture; (1) SARS-CoV spike; (2) MERS-CoV spike; (3) hCoV-229E spike; (4) hCoV-OC43 spike; (5) SARS-CoV-2 spike; (6) hCoV-HKU1 spike; and (7) hCoV-NL63 spike. [file elife-70330-fig2-figsupp5-data1.zip › Gel 1 raw.tif]

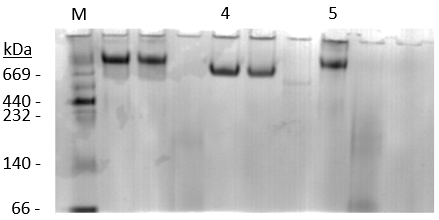

Supplement: Figure 2—figure supplement 5—source data 1. — The numbers match with the numbers in Figure 2—figure supplement 5: (M) Marker HMW-Native Protein Mixture; (1) SARS-CoV spike; (2) MERS-CoV spike; (3) hCoV-229E spike; (4) hCoV-OC43 spike; (5) SARS-CoV-2 spike; (6) hCoV-HKU1 spike; and (7) hCoV-NL63 spike. [file elife-70330-fig2-figsupp5-data1.zip › Gel 2 labeled.png]

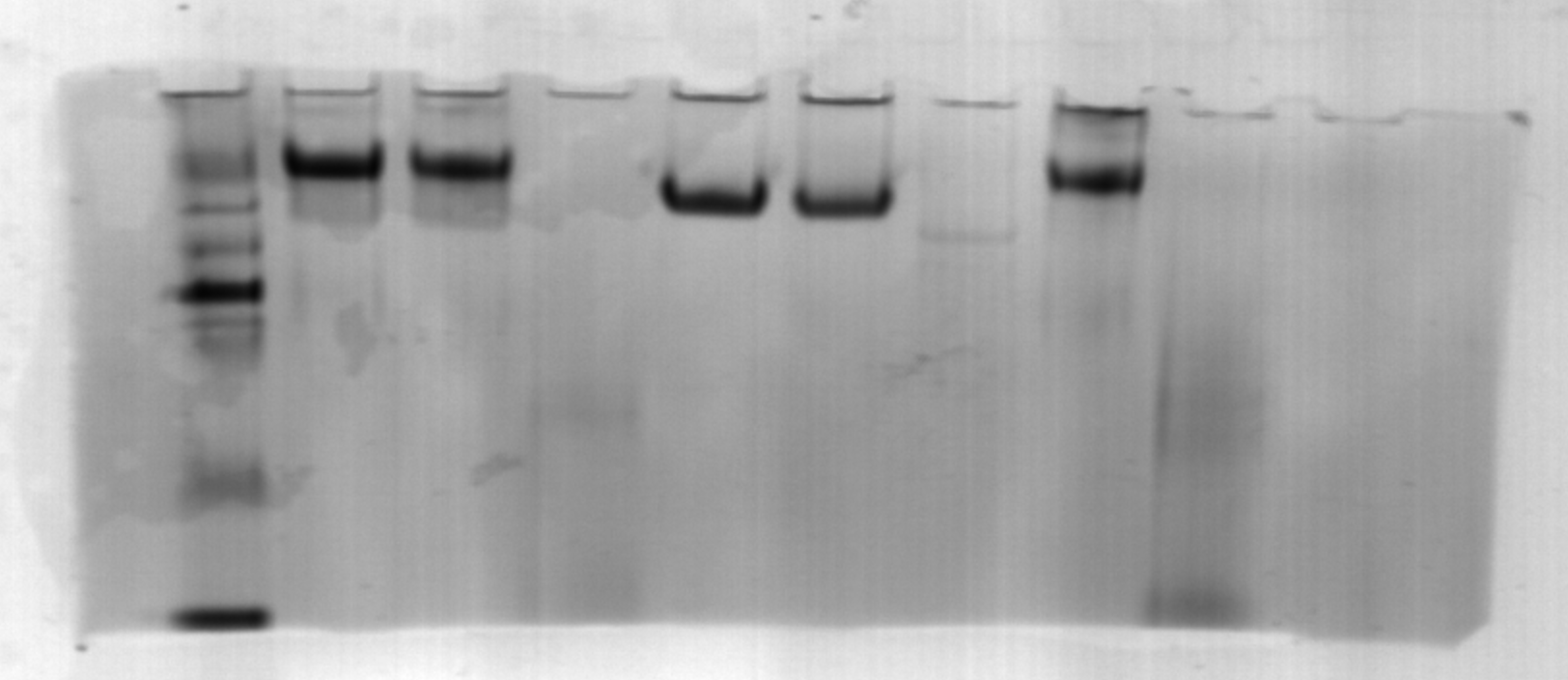

Supplement: Figure 2—figure supplement 5—source data 1. — The numbers match with the numbers in Figure 2—figure supplement 5: (M) Marker HMW-Native Protein Mixture; (1) SARS-CoV spike; (2) MERS-CoV spike; (3) hCoV-229E spike; (4) hCoV-OC43 spike; (5) SARS-CoV-2 spike; (6) hCoV-HKU1 spike; and (7) hCoV-NL63 spike. [file elife-70330-fig2-figsupp5-data1.zip › Gel 2 raw.tif]

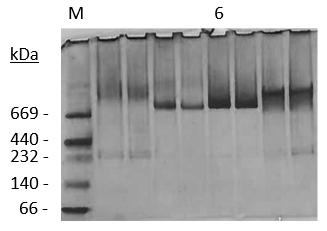

Supplement: Figure 2—figure supplement 5—source data 1. — The numbers match with the numbers in Figure 2—figure supplement 5: (M) Marker HMW-Native Protein Mixture; (1) SARS-CoV spike; (2) MERS-CoV spike; (3) hCoV-229E spike; (4) hCoV-OC43 spike; (5) SARS-CoV-2 spike; (6) hCoV-HKU1 spike; and (7) hCoV-NL63 spike. [file elife-70330-fig2-figsupp5-data1.zip › Gel 3 labeled.png]

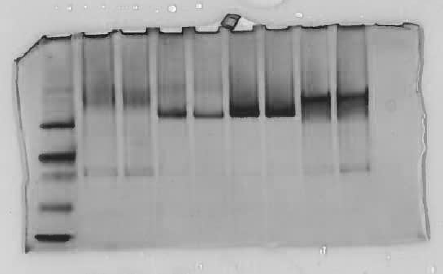

Supplement: Figure 2—figure supplement 5—source data 1. — The numbers match with the numbers in Figure 2—figure supplement 5: (M) Marker HMW-Native Protein Mixture; (1) SARS-CoV spike; (2) MERS-CoV spike; (3) hCoV-229E spike; (4) hCoV-OC43 spike; (5) SARS-CoV-2 spike; (6) hCoV-HKU1 spike; and (7) hCoV-NL63 spike. [file elife-70330-fig2-figsupp5-data1.zip › Gel 3 raw.png]

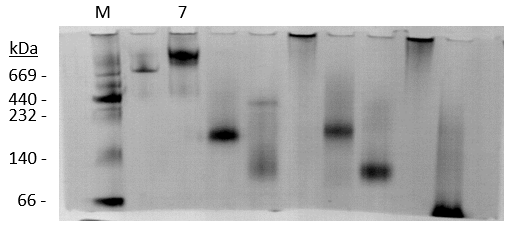

Supplement: Figure 2—figure supplement 5—source data 1. — The numbers match with the numbers in Figure 2—figure supplement 5: (M) Marker HMW-Native Protein Mixture; (1) SARS-CoV spike; (2) MERS-CoV spike; (3) hCoV-229E spike; (4) hCoV-OC43 spike; (5) SARS-CoV-2 spike; (6) hCoV-HKU1 spike; and (7) hCoV-NL63 spike. [file elife-70330-fig2-figsupp5-data1.zip › Gel 4 labeled.png]

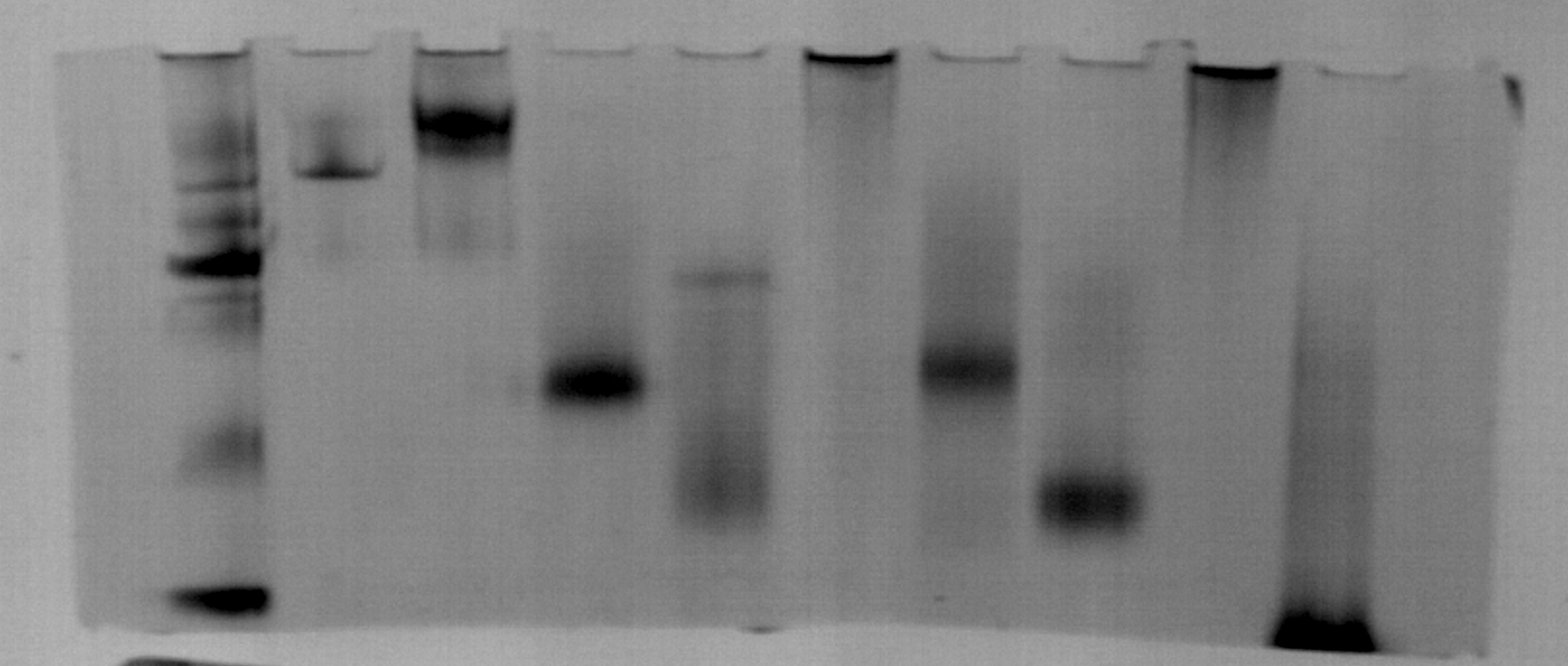

Supplement: Figure 2—figure supplement 5—source data 1. — The numbers match with the numbers in Figure 2—figure supplement 5: (M) Marker HMW-Native Protein Mixture; (1) SARS-CoV spike; (2) MERS-CoV spike; (3) hCoV-229E spike; (4) hCoV-OC43 spike; (5) SARS-CoV-2 spike; (6) hCoV-HKU1 spike; and (7) hCoV-NL63 spike. [file elife-70330-fig2-figsupp5-data1.zip › Gel 4 raw.tif]
