# Supplementary material for: Cross-reactive antibodies after SARS-CoV-2 infection and vaccination
Source: eLife. 2021 Nov 23;10:e70330. doi: 10.7554/eLife.70330 (PMC8610423; doi:10.7554/eLife.70330)
Supplement: Supplementary file 1. [file elife-70330-supp1.docx]

**Supplementary File 1. Sociodemographics, clinical characteristics and severity scoring for COVID-19 patients**

| Sociodemographics and  Clinical Characteristics | Number of patients  n (%) |
| --- | --- |
| Sex |  |
| Male | 27 (54) |
| Female | 23 (46) |
| Age (years) |  |
| <35 | 12 (24) |
| 35-60 | 21 (42) |
| >60 | 17 (34) |
| Hospitalization |  |
| Home | 25 (50) |
| Admission | 25 (50) |
| Disease severity | |
| 0 | 0 (0) |
| 1 | 16 (32) |
| 2 | 17 (34) |
| 3 | 14 (28) |
| 4 | 3 (6) |
| Serological positive (WANTAI) | |
|  | 49 (98) |

| **Score** | **Short name** | **Criteria** |
| --- | --- | --- |
| 0 | Asymptomatic | SARS-CoV-2 proven infection without developing symptoms of COVID-19 |
| 1 | Mild | Symptoms of COVID-19 without evidence of viral pneumonia or hypoxia |
| 2 | Moderate | Clinical signs of pneumonia but no signs of severe pneumonia, including SpO2 ≥ 90% on room air |
| 3 | Severe | Clinical signs of severe pneumonia: respiratory rate > 30 breaths/min, spO2 <90% on room air |
| 4 | Critical | ICU admission (ARDS, Sepsis, Septic shock) |
