# Supplementary material for: Cross-reactive antibodies after SARS-CoV-2 infection and vaccination
Source: eLife. 2021 Nov 23;10:e70330. doi: 10.7554/eLife.70330 (PMC8610423; doi:10.7554/eLife.70330)
Supplement: Supplementary file 2. — Sequence identity matrices were composed of all coronavirus spike proteins in this study. All sequences comprise only the truncated ectodomain of each spike as was used to generate the recombinant proteins. S1, S2, and RBD were defined as noted in the corresponding GenBank sequences (see Materials and methods). Multiple sequence alignments were performed and sequence identities calculated using Clustal Omega 1.2.4. [file elife-70330-supp2.docx]

**Supplementary File 2. Sequence identity matrices for the ectodomains of all hCoV S proteins**

| **S** |  | |  | |  | |  | |  | |  | |  | |  |
| --- | --- | --- | --- | --- | --- | --- | --- | --- | --- | --- | --- | --- | --- | --- | --- |
|  | SARS 2 S | | SARS S | | MERS S | | OC43 S | | HKU1 S | | 229E S | | NL63 S | |  |
| SARS 2 S | 100% | |  | |  | |  | |  | |  | |  | |  |
| SARS S | 75,4% | | 100% | |  | |  | |  | |  | |  | |  |
| MERS S | 30,9% | | 31,4% | | 100% | |  | |  | |  | |  | |  |
| OC43 S | 30,0% | | 30,6% | | 31,7% | | 100% | |  | |  | |  | |  |
| HKU1 S | 30,3% | | 31,1% | | 32,2% | | 65,9% | | 100% | |  | |  | |  |
| 229E S | 27,4% | | 27,2% | | 28,4% | | 27,7% | | 28,8% | | 100% | |  | |  |
| NL63 S | 26,2% | | 24,8% | | 27,1% | | 28,1% | | 28,3% | | 65,1% | | 100% | |  |
| **S1** |  | |  | |  | |  | |  | |  | |  | |  |
|  | SARS 2 S1 | | SARS S1 | | MERS S1 | | OC43 S1 | | HKU1 S1 | | 229E S1 | | NL63 S1 | |  |
| SARS 2 S1 | 100% | |  | |  | |  | |  | |  | |  | |  |
| SARS S1 | 66,8% | | 100% | |  | |  | |  | |  | |  | |  |
| MERS S1 | 21,0% | | 22,1% | | 100% | |  | |  | |  | |  | |  |
| OC43 S1 | 25,1% | | 24,7% | | 23,4% | | 100% | |  | |  | |  | |  |
| HKU1 S1 | 23,9% | | 25,2% | | 26,4% | | 61,5% | | 100% | |  | |  | |  |
| 229E S1 | 17,5% | | 16,9% | | 18,1% | | 16,5% | | 19,4% | | 100% | |  | |  |
| NL63 S1 | 16,4% | | 14,6% | | 15,1% | | 16,9% | | 17,5% | | 51,7% | | 100% | |  |
| **S2** | |  | |  | |  | |  | |  | |  | |  | |
|  | | SARS 2 S2 | | SARS S2 | | MERS S2 | | OC43 S2 | | HKU1 S2 | | 229E S2 | | NL63 S2 | |
| SARS 2 S2 | | 100% | |  | |  | |  | |  | |  | |  | |
| SARS S2 | | 87,6% | | 100% | |  | |  | |  | |  | |  | |
| MERS S2 | | 44,5% | | 43,4% | | 100% | |  | |  | |  | |  | |
| OC43 S2 | | 41,8% | | 40,9% | | 45,2% | | 100% | |  | |  | |  | |
| HKU1 S2 | | 41,3% | | 40,6% | | 43,5% | | 71,7% | | 100% | |  | |  | |
| 229E S2 | | 38,0% | | 37,7% | | 38,0% | | 35,0% | | 35,0% | | 100% | |  | |
| NL63 S2 | | 38,2% | | 35,9% | | 36,9% | | 36,6% | | 35,4% | | 77,7% | | 100% | |
| **RBD** | |  | |  | |  | |  | |  | |  | |  | |
|  | | SARS 2 RBD | | SARS RBD | | MERS RBD | | OC43 RBD | | HKU1 RBD | | 229E RBD | | NL63 RBD | |
| SARS 2 RBD | | 100% | |  | |  | |  | |  | |  | |  | |
| SARS RBD | | 73,4% | | 100% | |  | |  | |  | |  | |  | |
| MERS RBD | | 19,2% | | 21,3% | | 100% | |  | |  | |  | |  | |
| OC43 RBD | | 23,1% | | 24,6% | | 27,1% | | 100% | |  | |  | |  | |
| HKU1 RBD | | 19,7% | | 23,4% | | 27,5% | | 60,0% | | 100% | |  | |  | |
| 229E RBD | | 16,7% | | 12,8% | | 23,6% | | 17,1% | | 21,1% | | 100% | |  | |
| NL63 RBD | | 12,5% | | 17,0% | | 20,0% | | 17,1% | | 15,6% | | 57,3% | | 100% | |

Sequence identity matrices were composed of all coronavirus spike proteins in this study. All sequences comprise only the truncated ectodomain of each spike as was used to generate the recombinant proteins. S1, S2 and RBD were defined as noted in the corresponding Genbank sequences (see methods). Multiple sequence alignments were performed and sequence identities calculated using Clustal Omega 1.2.4.
